# Supplementary material for: Intranasal Treatment of Ferrets with Inert Bacterial Spores Reduces Disease Caused by a Challenging H7N9 Avian Influenza Virus
Source: Vaccines (Basel). 2022 Sep 19;10(9):1559. doi: 10.3390/vaccines10091559 (PMC9502451; doi:10.3390/vaccines10091559)
Supplement: Supplementary file 1 [file vaccines-10-01559-s001.zip › Figure S1.pdf]

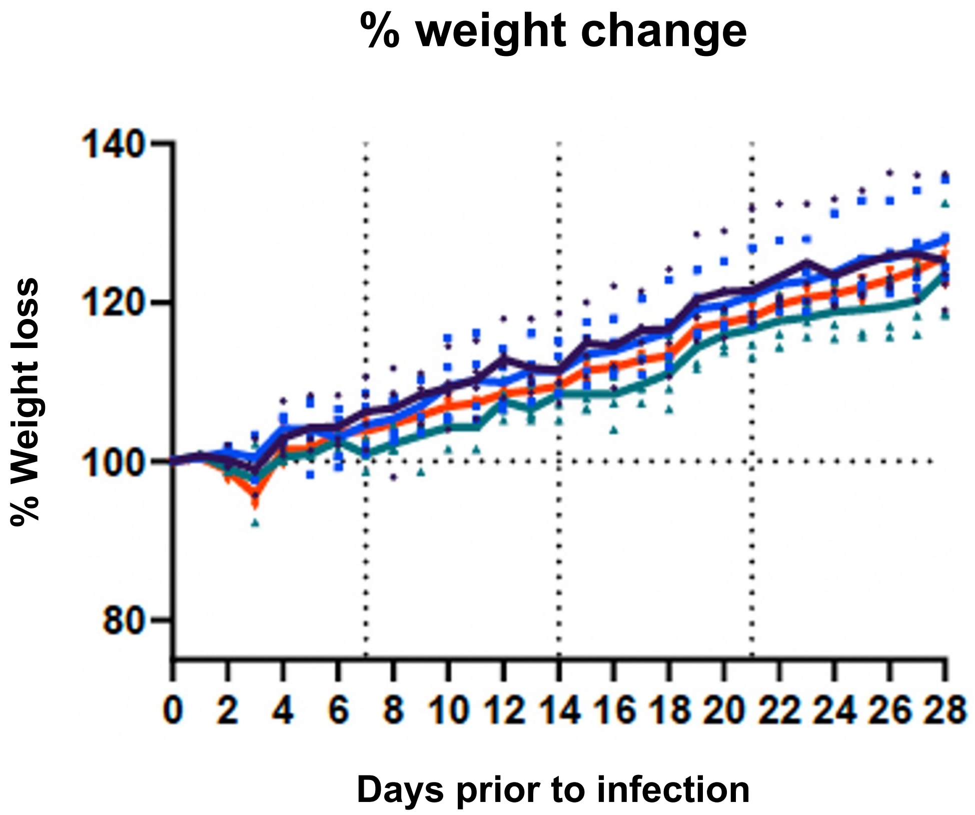

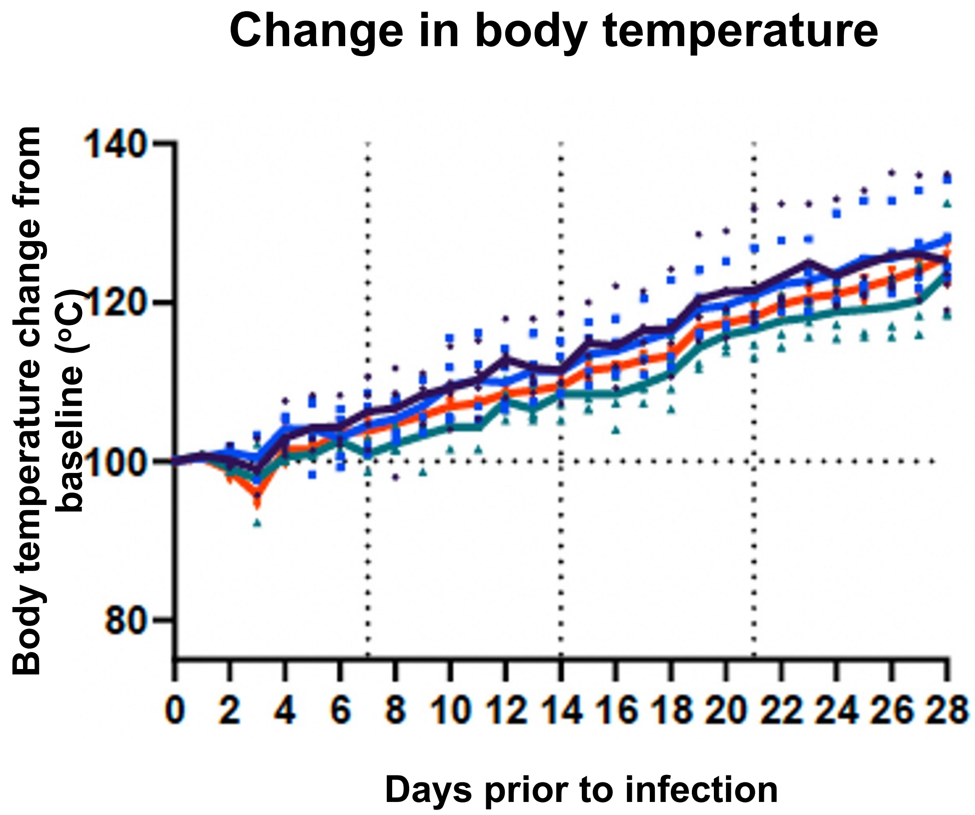

Supplementary Figure S1. Clinical signs, change in body temperature and weight loss pre-H7N9 infection

Ferrets were treated with 0.5ml of DSM 32444^{K} or PBS on four separate occasions, 7-days apart. Individual values were plotted per animal and lines indicate the mean values per group sorted by sex. (**left**) weights and (**right**) body temperatures were taken at 1 day prior to intranasal dosing, which was used to assess a baseline, and then daily following dosing with DSM 32444^{K} or PBS until day after which animals were challenged with H7N9. Statistical significance determined using the Holm-Sidak method, with alpha = 0.05. Key: Black line, Control Males; Blue line, Control females; Green line, DSM 32444^{K}-treated males; Red line, DSM 32444^{K}-treated females.
