# Supplementary material for: Intranasal Treatment of Ferrets with Inert Bacterial Spores Reduces Disease Caused by a Challenging H7N9 Avian Influenza Virus
Source: Vaccines (Basel). 2022 Sep 19;10(9):1559. doi: 10.3390/vaccines10091559 (PMC9502451; doi:10.3390/vaccines10091559)
Supplement: Supplementary file 1 [file vaccines-10-01559-s001.zip › Table S1.pdf]

Supplementary Table S1. Clinical Score Sheet used for ferrets infected with Influenza A viruses

No	Parameter	Criteria	Score
1	*Alertness*	attentive (curious, alert)	0
		slight reduced - hesitant, disinterested	1
		inactive, gets up only when stimulated, lies down again	2
		recumbent, won't get up when stimulated	3
2	*Weight loss*	no significant weight loss 0-5%	0
		change from baseline weight between 5-10%	1
		change from baseline weight between 10-15%	2
		change from baseline weight over 15%	3
3	*Body tension*	relaxed	0
		slightly hunched back	1
		flaccid or hunched and rigid body	2
4	*Breathing*	normal	0
		sneezing/coughing	1
		intermittent breathing difficulties, or increase/decrease in respiratory weight	2
		persistent breathing distress, wheezing	3
5	*Coat/skin*	smooth, flat coat	0
		slightly roughened coat, lack of grooming<br>slight reddening or oedema at vaccination site	1
		rough dull looking coat<br>moderate reddening or oedema at vaccination site	2
6	*Eyes*	eyes bright and clear	0
		clear discharge from eyes/nose	1
		conjunctivitis, yellow discharge from eyes and nose	2
7	*Appetite/drinking*	normal, eats most of food offered	0
		little food eaten, shows interest in treats but doesn't fully eat them	1
		shows no interest in food or treats, nothing eaten or drunk	2
8	*Temperature*	<1°C (±) change from baseline	0
		+1-1.5°C change from baseline	1
		more than +1.5°C change from baseline	2
		> 41.0°C or decrease of 1°C within 24h	3
	Total clinical score	Out of a theoretical score of 20	
